# Lithotomy Performed Twice in Three Days on the Same Patient

**Published:** 1829-01

**Authors:** M. Dupuytren


### LITHOTOMY.

*Lithotomy performed twice in three days on the same Patient, by* M. Dupuytren. From a Correspondent at Paris.

On the 17th November, an old man was brought into the theatre of the Hôtel Dieu, for the extraction of a calculus, which was followed by one of those disastrous results that occasionally fall to the lot of the most eminent practitioners. The subject of this notice did not, indeed, die *under* the knife; though, after long protracted but judicious efforts on the part of M. Dupuytren, the stone remained the first day immovably fixed in the bladder. On the third day, a second and successful attempt was made to extract the stone by the recto-vesical method; but the unfortunate patient expired in the course of the night.

Few cases offer more points of instruction to the practitioner than this. The cause of the difficulty was not obscure, but, as it frequently happens, was not detected till it was too late to be remedied the first day; since the patient's state made it then necessary to remove him to his bed, and to defer all further measures to a future day, if he should indeed survive the consequences of the violent irritation and torture which he had already undergone.

It seems hardly necessary to suggest that the only impediment to the exit of a stone, under the hands of so distinguished an operator as M. Dupuytren, must have arisen solely from its extraordinary bulk, and the want of proportionate space for its exit. It will be found, however, that the judgment of the surgeon in the adoption of his method on this occasion, was not altogether free from blame. The size of the stone had been previously ascertained by the introduction of a finger into the rectum, and the application of the hand to the hypogastric region. These were separated by a resisting body for the space of two inches and a half, so that the larger diameter of the stone, and perhaps the smaller one, equalled the usual distance between the tuberosities of the ischia, which is the largest opening through which the stone could be extracted. By a sort of fatality, however, the diverging of the two bones in this patient was less than usual.

The entrance of the staff into the bladder was obstructed by a sonorous body impacted in the neck of this viscus. The sound, on percussion, was audible in the back seats of the theatre; and so completely did the stone fill the cavity of the bladder, that not a particle of urine was retained, and a urinal was constantly worn to receive it *guttatim.* The patient complained of uneasiness in the kidneys, of considerable pain in the bladder, which extended to the glans, where the usual sensation of itching was felt. The disease had, in its present form, existed for ten years; and had been preceded by the discharge of gravel, and occasionally small calculi, through the urethra. At length one became impacted, was broken, and removed with considerable pain. When the gravel ceased to be discharged, the calculus began to form; a fact which M. Dupuytren noted as one of constant occurrence, and the cause of which may be readily conceived.

It was obvious that no ordinary incision could liberate a stone of such magnitude, and the space under the arch of the pubis was evidently not sufficient. The lateral operation was therefore out of the question; and the unnatural approximation of the tuberosities of the ischia in this subject was not very favorable to the recto-vesical incision. Yet this, or the hypogastric, was the method peculiarly called for under similar circumstances, and no alternative remained but to choose between them.

The expediency of lithotritic was cursorily discussed; but, as the principal impediments to success, a stone of considerable size and an irritable and contracted bladder, existed, the negative was immediately pronounced. The difficulty of grasping the calculus by the *litholabe* would have been insurmountable; and, were it otherwise, the numerous operations that would be requisite for the complete perforation and destruction of such a stone would alone have been sufficient to cause its rejection.

Although an incision above the pubes affords an easy exit for the stone, yet this method is frequently followed by infiltration of urine into the cellular membrane interposed between the abdominal muscles, and thus causes peritoneal inflammation, gangrene, and death. Besides, an impediment might arise from the state of the bladder in this patient: for the extreme, nay invincible, difficulties which have been sometimes experienced in endeavouring to distend a cartilaginous bladder, so as to make it rise above the pubes, might occur in this case; for a bladder, thus diseased and irritable, would not yield in the slightest degree, and the agony of the patient must compel the surgeon to abandon the attempt. A staff is generally introduced to carry the bladder above the pubes; but here it could not pass at the anterior part, and it became necessary to use one of small size, and slightly curved for the space of an inch at its extremity, for the purpose of passing beyond the stone, and of conducting the knife during the operation that was ultimately chosen.

But is the recto-vesical method free from objection and danger? Not altogether. It is followed occasionally by inflammation of the cellular membrane within the pelvis, and sometimes by recto-vesical fistulæ. The mucous membrane of the rectum, unaccustomed to the irritation of urine, might become inflamed by contact with it. The vas deferens is liable to be injured. "But how," says M. D. "can these disadvantages be compared with the dangers of the hypogastric operation, which some are disposed to recommend at the present day, and which nevertheless has been abandoned. It is an undoubted fact that, as often as surgeons have thought proper to renew the attempt, more patients fall victims to the hypogastric than to the perineal incision."

On a balance of evils, M. D. inclined to the recto-vesical method. If fistulæ should occur, he conceived it would be less disastrous than peritonitis; nor would the inflammation of the cellular membrane within the pelvis be so likely to occur as that of the peritoneum.

Nothing could be more judicious than this reasoning; and, though the concluding sentence of an excellent clinical lecture had but just escaped from the lips of M. Dupuytren as the patient was placed upon the table, and prepared for the operation, yet in this short interval an unlucky train of thought, it seems, subverted this decision, and induced him to do that which for three quarters of an hour he had shown to be in the present case objectionable.

*The bilateral operation was performed*, which differs from that originally proposed only in the *external incision!* This was made perpendicularly in the raphe down to the anus, and the double-bladed bistouri caché was used to divide the bladder and prostate on both sides.

The incisions being lateral instead of posterior, it became impossible to draw the stone in a direction perpendicular to the axis of the pelvis. After long-continued efforts and occasional repose for deliberation, it was found that the stone was unlikely to move; and it became a question whether recourse should be had to the hypogastric operation or to the recto-vesical, or whether the stone should be broken in situ, not by lithotritic, but by mechanical power, which from time immemorial has been recommended in all cases where the stone has been too large to pass either through the incision or through the natural aperture of the pelvis. However, it was deemed advisable to defer all other proceedings at present, and the patient was conveyed back to his bed, in a situation which has been recommended in all cases by those surgeons who, like DESCHAMPS, advise dividing the operation into two distinct periods; a measure, in truth, absurd, and which nothing but unforeseen and pressing events can justify.

The state of the patient, at the close of this calamitous event, was not unlike that of a child operated upon by FRANCO in the middle of the sixteenth century, and to which we are indebted for the high operation. Had the latter been performed on the present occasion, the resemblance would have been perfect. As the stone in the child had resisted the most persevering efforts for its removal, the surgeon was entreated by the parents to desist, and to abandon the little sufferer to his fate. But, as he states, "*being desirous of avoiding the reproach of having failed,*" (a laudable motive!) he introduced his finger into the rectum, projected the stone above the pubis, and extracted it through an incision made into the bladder. The child recovered, but was extremely ill, and the operator had not sufficient confidence in this method to advise its adoption. It remained forgotten or neglected until about twenty years afterwards, when it was brought into notice by ROUSSET.

M. Dupuytren was evidently distressed at the result; his usual firmness abandoned him, and his countenance betrayed the conflict that was passing within. He immediately explained to those near him the error which he had committed, and on the following morning publicly acknowledged the same to the assembled practitioners and students. How praiseworthy is this candour, and how beneficial to science! How much more do we learn by a cool impartial consideration of occasional errors, than by the ordinary course of unruffled practice. How injurious the notion that the reputation of a man of science can lose from such voluntary disclosures! Can the great and well-merited fame of M. Dupuytren suffer? Certainly not. Enthusiastic as he is in the pursuit of professional knowledge, ever intent upon extending the boundaries of our art, and anxious to relieve the sufferings of humanity, where is the being so malignant or so daring as to utter a word of reprobation, if, in the fluctuating distractions of his mind, in estimating the comparative merits of various methods, his choice should be sometimes erroneous.

On the morning after the operation, no bad symptom had been experienced. The patient had been frequently put into the warm bath, where he remained from one to two hours at a time, according to his feelings. He was twice bled, and leeches had been applied to the buttocks. As the median incision divides no vessels of importance, no hemorrhagy had taken place; the pulse was rather calm; no shiverings had been experienced; no pain or uneasiness from pressing over the bladder: yet the blood was decidedly buffy, although not to a great depth. It was remarked that the patient experienced less pain in voiding the urine than before the operation.

At the close of the second day, pain was experienced in the left iliac region: fear was entertained that inflammation had seized the cellular membrane within the pelvis. No soreness on pressing the abdomen, to which cataplasms had been applied through the day, excepting during the use of the warm bath.

Third day, symptoms seemed aggravated. He had been at intervals several hours in the warm bath, and leeches had been applied in large numbers. The abdomen was distended with wind, accompanied by constant desire to go to stool. This was supposed to arise from the pressure of the stone on the rectum.

In the evening, the sufferings of the patient were increased. The stone had partly descended into the wound. An incision was made through the sphincter ani, as in the recto-vesical operation, and, after some difficulty in getting the forceps to hold it, was at length withdrawn.

About midnight the man died.

---

